# More Than Bone Health: The Many Roles for Vitamin D

**DOI:** 10.3390/nu12082388

**Published:** 2020-08-10

**Authors:** Emma Beckett

**Affiliations:** 1 School of Environmental and Life Science, The University of Newcastle, Ourimbah, NSW 2258, Australia; Emma.Beckett@newcastle.edu.au; 2 Australia and Hunter Medical Research Institute, New Lambton Heights, NSW 2305, Australia

Vitamin D is well known for its important roles in maintaining calcium homeostasis and bone mineralization via the regulation of calcium mobilization and renal reabsorption, and the intestinal absorption of both calcium and phosphorus [1]. These functions have been established for many decades. In fact, the associations between vitamin D-containing foods, sun exposure and bone health were identified over a century ago, long before vitamin D itself, the mechanism for its synthesis and its active metabolite were discovered [2,3]. However, as outlined by Carsten Carlberg in a recent review in *Nutrients* [4], this classical focus on bone health presents a narrow view of the functions of vitamin D. With the development of the field of nutrigenomics, it is now becoming clear that the active vitamin D metabolite, calcitriol, has many more physiological functions. 

Nutrigenomics describes the role of nutrients and bioactive compounds in foods in controlling gene expression and, consequently, on the proteome and the metabolome [5]. The vitamin D receptor (VDR) is a widely expressed member of the nuclear receptor superfamily. When calcitriol binds to the cytosolic VDR phosphorylation, and heterodimerization with the retinoic X receptor (RXR) occurs, the complex translocates to the nucleus [6,7]. The complex then binds with the vitamin D response element (VDRE) as a transcription factor, with binding leading to conformation change from repression to activation via the recruitment of transcription co-activators [6,7], ultimately leading to gene expression. 

As such, VDR, like other nutrient-sensing members of the nuclear receptor superfamily, allows gene expression in many cell types to adapt with nutritional status [4]. The VDR is highly expressed on many cell and tissue types involved in the classical roles of vitamin D, including bone, kidneys, liver and intestines [8], and there are overlaps between its traditional and genomic roles. However, the VDR is also expressed in a wide variety of cell and tissue types, including those not involved in calcium regulation, such as dermal and immune cells, cardiovascular tissues and in the nervous system [9]. Transcriptome-wide analyses suggest that via VDR–VDRE interactions, 1,25(OH)_2_D can regulate the expression of hundreds of genes, and it has become clear that this includes many genes not related to mineral regulation [10]. 

The nutrigenomic functions of vitamin D may also complement or interact with its epigenetic functions [4]. These mechanisms include DNA methylation, and remodelling of chromatin and histones, which influence accessibility to genes [11]. Therefore, they play important roles in regulating gene expression indirectly without changes to the genome. VDR binds to a genomic region only when it finds its preferred VDRE sequence motifs within an accessible chromatin. As such, vitamin D, like other epigenetically active micro- and macronutrients, may influence the accessibility of VDRE in a transient and reversible way, thus further regulating gene expression [11,12]. This may act as a modifiable molecular regulator to maintain homeostasis in response to changing light exposure or intake levels [13].

Through nutrigenomic and epigenomic functions, calcitriol and VDR are implicated in the modulation of risk for a variety of diseases. There is emerging in vitro and in vivo evidence that this system is involved in modulation of innate and adaptive immune responses, cell differentiation, reactive oxygen species detoxification, apoptosis, angiogenesis and invasion in various cell types [14,15,16]. These functions have consequences for tumorigenesis, metastasis, immune function and cardiovascular disease signalling pathways [17,18]. Roles are also suggested for calcitriol and VDR in neurological and neuromuscular disorders, with vitamin D metabolites able to cross the blood–brain barrier [19]. While many candidate genes and common pathways such as the Wnt signalling and NF-κB pathways have been identified as responsive to calcitriol, more work is needed to elucidate the precise mechanisms involved [17]. 

The genomic impacts of calcitriol may vary between individuals due to nutrigenetics. Nutrigenetics describes the role of gene variants in modifying responses in metabolism and detection of nutrients [20]. Polymorphisms in the VDR gene [21], the vitamin D binding protein [22] and the genes for the hydroxylase enzymes involved in vitamin D metabolism [23] can lead to differential responses to the same stimuli. Large ranges in the individual responses to vitamin D supplementation have been demonstrated in multiple studies [22,24], with these responses common to genotypic groups [22,24]. Evolutionary nutrigenetic pressures are believed to have influenced the differential development of skin pigmentation in populations from different latitudes to maintain nutrient homeostasis, including that of vitamin D. However, associations may extend out of the vitamin D system [25]. Polymorphism in the VDR and hydroxylase genes involved in the metabolism of vitamin D have also been associated with the modulation of risk for autoimmune diseases, some cancers and bone and muscle health [21,23]. 

As genomic techniques become more powerful and affordable, opportunities are expanding to uncover vast amounts of data on vitamin D-regulated epigenome and transcriptome changes in a range of cellular systems and disease states. This will allow the uncovering of the mechanisms responsible for the observed associations with disease. It is hoped that with the further elucidation of the genomic mechanisms involved that eventually vitamin D may be incorporated into prevention or treatment strategies for many conditions, in addition to bone health. This may be most beneficial when combined with personalized nutrition approaches to account for the differential responses in individuals with key vitamin D-related variants. 

## References

[1] DeLuca H.F. (1986). The metabolism and functions of vitamin D. Adv. Exp. Med. Biol..

[2] O’Riordan J.L.H., Bijvoet O.L.M. (2014). Rickets before the discovery of vitamin D. Bonekey Rep..

[3] Deluca H.F. (2014). History of the discovery of vitamin D and its active metabolites. Bonekey Rep..

[4] Carlberg C. (2019). Nutrigenomics of Vitamin D. Nutrients.

[5] Ferguson L.R. (2009). Nutrigenomics approaches to functional foods. J. Am. Diet. Assoc..

[6] Pike J.W., Meyer M.B. (2014). Fundamentals of vitamin D hormone-regulated gene expression. J. Steroid Biochem. Mol. Biol..

[7] Carlberg C., Campbell M.J. (2013). Vitamin D receptor signaling mechanisms: Integrated actions of a well-defined transcription factor. Steroids.

[8] Haussler M.R., Jurutka P.W., Hsieh J.C., Thompson P.D., Selznick S.H., Haussler C.A., Whitfield G.K. (1995). New understanding of the molecular mechanism of receptor-mediated genomic actions of the vitamin D hormone. Bone.

[9] Wang Y., Zhu J., DeLuca H.F. (2012). Where is the vitamin D receptor?. Arch. Biochem. Biophys..

[10] Pike J.W., Meyer M.B., Lee S.-M., Onal M., Benkusky N.A. (2017). The vitamin D receptor: Contemporary genomic approaches reveal new basic and translational insights. J. Clin. Investig..

[11] Fetahu I.S., Höbaus J., Kállay E. (2014). Vitamin D and the epigenome. Front. Physiol..

[12] Carlberg C. (2017). Molecular endocrinology of vitamin D on the epigenome level. Mol. Cell. Endocrinol..

[13] Beckett E.L., Jones P., Veysey M., Duesing K., Martin C., Furst J., Yates Z., Jablonski N.G., Chaplin G., Lucock M. (2017). VDR gene methylation as a molecular adaption to light exposure: Historic, recent and genetic influences. Am. J. Hum. Biol..

[14] Borradale D., Kimlin M. (2009). Vitamin D in health and disease: An insight into traditional functions and new roles for the ‘sunshine vitamin’. Nutr. Res. Rev..

[15] Prietl B., Treiber G., Pieber T.R., Amrein K. (2013). Vitamin D and immune function. Nutrients.

[16] Davis C.D., Milner J.A. (2011). Nutrigenomics, Vitamin D and Cancer Prevention. Lifestyle Genom..

[17] Gil A., Plaza-Diaz J., Mesa M.D. (2018). Vitamin D: Classic and Novel Actions. Ann. Nutr. Metab..

[18] Charoenngam N., Holick M.F. (2020). Immunologic Effects of Vitamin D on Human Health and Disease. Nutrients.

[19] Orme R.P., Middleditch C., Waite L., Fricker R.A. (2016). The Role of Vitamin D₃ in the Development and Neuroprotection of Midbrain Dopamine Neurons. Vitam. Horm..

[20] Farhud D., Zarif Yeganeh M., Zarif Yeganeh M. (2010). Nutrigenomics and nutrigenetics. Iran. J. Public Health.

[21] Valdivielso J.M., Fernandez E. (2006). Vitamin D receptor polymorphisms and diseases. Clin. Chim. Acta.

[22] Al-Daghri N.M., Mohammed A.K., Bukhari I., Rikli M., Abdi S., Ansari M.G.A., Sabico S., Hussain S.D., Alenad A., Al-Saleh Y. (2019). Efficacy of vitamin D supplementation according to vitamin D-binding protein polymorphisms. Nutrition.

[23] Hu Z., Tao S., Liu H., Pan G., Li B., Zhang Z. (2019). The Association between Polymorphisms of Vitamin D Metabolic-Related Genes and Vitamin D_3_ Supplementation in Type 2 Diabetic Patients. J. Diabetes Res..

[24] Saksa N., Neme A., Ryynänen J., Uusitupa M., de Mello V.D.F., Voutilainen S., Nurmi T., Virtanen J.K., Tuomainen T.-P., Carlberg C. (2015). Dissecting high from low responders in a vitamin D3 intervention study. J. Steroid Biochem. Mol. Biol..

[25] Jones P., Lucock M., Veysey M., Beckett E. (2018). The Vitamin D—Folate Hypothesis as an Evolutionary Model for Skin Pigmentation: An Update and Integration of Current Ideas. Nutrients.

